# Regulatory and sequence evolution in response to selection for improved associative learning ability in *Nasonia vitripennis*

**DOI:** 10.1186/s12864-018-5310-9

**Published:** 2018-12-10

**Authors:** Ken Kraaijeveld, Vicencio Oostra, Maartje Liefting, Bregje Wertheim, Emile de Meijer, Jacintha Ellers

**Affiliations:** 1 Department of Ecological Science, Faculty of Earth and Life Sciences, Vrije Universiteit, De Boelelaan 1085, 1081 HV Amsterdam, The Netherlands; 2 Department of Genetics, Evolution and Environment, University College London, Darwin Building, Gower Street, WC1E 6BT London, UK; 3 Groningen Institute for Evolutionary Life Sciences, University of Groningen, Groningen, The Netherlands; 4 Leiden Genome Technology Center, Department of Human Genetics, Leiden University Medical Center, Leiden, The Netherlands

**Keywords:** Memory retention, *Nasonia vitripennis*, Artificial selection, Polygenic adaptation, Pooled sequencing, Complex trait, Expression

## Abstract

**Background:**

Selection acts on the phenotype, yet only the genotype is inherited. While both the phenotypic and genotypic response to short-term selection can be measured, the link between these is a major unsolved problem in evolutionary biology, in particular for complex behavioural phenotypes.

**Results:**

Here we characterize the genomic and the transcriptomic basis of associative learning ability in the parasitic wasp *Nasonia vitripennis* and use gene network analysis to link the two. We artificially selected for improved associative learning ability in four independent pairs of lines and identified signatures of selection across the genome. Allele frequency diverged consistently between the selected and control lines in 118 single nucleotide polymorphisms (SNPs), clustering in 51 distinct genomic regions containing 128 genes. The majority of SNPs were found in regulatory regions, suggesting a potential role for gene expression evolution. We therefore sequenced the transcriptomes of selected and control lines and identified 36 consistently differentially expressed transcripts with large changes in expression. None of the differentially expressed genes also showed sequence divergence as a result of selection. Instead, gene network analysis showed many of the genes with consistent allele frequency differences and all of the differentially expressed genes to cluster in a single co-expression network. At a functional level, both genomic and transcriptomic analyses implicated members of gene networks known to be involved in neural plasticity and cognitive processes.

**Conclusions:**

Taken together, our results reveal how specific cognitive abilities can readily respond to selection via a complex interplay between regulatory and sequence evolution.

**Electronic supplementary material:**

The online version of this article (10.1186/s12864-018-5310-9) contains supplementary material, which is available to authorized users.

## Background

Understanding the genetic basis of adaptive polygenic phenotypes is a major challenge in evolutionary biology, in particular for complex behavioural phenotypes. Aided by development of high-throughput sequencing technology, recent studies have made significant progress in identifying gene loci that shape polygenic phenotypes [1]. It has become clear that short-term responses to selection usually involve gene regulation, rather than changes in protein-coding sequences [2–7]. However, we currently have limited understanding of how these observed adaptive changes in gene expression and phenotype are specified by variation at genomic loci, which ultimately form the basis of inheritance. To understand how complex phenotypes evolve, it is therefore important to link transcriptomic and phenotypic changes to genomic changes during the same episode of adaptation.

A behavioural trait that responds readily to selection is associative learning ability and memory formation, which are part of an organism’s cognitive repertoire [8, 9]. Learning and memory enable an organism to use information from previous experience for a functional change in behaviour in response to changing situations [10]. This is specifically relevant when environments vary within the lifetime of an individual, as it enables the establishment of predictive relationships [11]. Most if not all animals studied have demonstrated the ability of at least a simple form of learning [12, 13], yet the diversity in cognitive abilities is enormous. Studies on a large number of species have revealed large intra- and interspecific variation in how quickly information can be learned and how long memory will last [14], even between closely related species [15, 16] or different populations of the same species [17]. It is commonly assumed that the wide variety in learning abilities reflect adaptations to the differing natural conditions under which these animals operate and that specific learning abilities are the net result of the costs and benefits of learning under the encountered conditions [18–21]. However, the genetic architecture that shapes natural variation in learning and memory dynamics remains poorly understood [22].

Studies using laboratory-generated mutants of *Drosophila melanogaster* (like e.g. *dunce* or *rutabaga*) have been successful in identifying single loci with large effects on memory formation [23–25]. It remains to be investigated how much the specific genes described for these mutants contribute to natural, segregating variation in learning ability and memory retention. Such variation in cognitive ability may very well depend on more subtle variation in the genes described in these mutants or a wide range of completely different genes that are important for underlying neural pathways or processes, like e.g. the many genes associated with the dopaminergic neural circuitry [26] or neural plasticity in brain cells that influence approach or avoidance behaviour [27]. In addition, focusing on sequence evolution of target genes neglects processes of regulatory evolution (e.g. gene expression) and its contribution to the evolution of adaptive phenotypes. For example, induced expression of a specific splice variant of cAMP-responsive transcription factor CREB in *D. melanogaster* results in long-term memory formation after a single conditioning trial, whereas this normally requires 10 spaced conditioning trials [28]. A GWAS study on educational attainment in humans localised a disproportionate number of SNPs in regions that regulate brain-specific gene expression or regions associated with histones marked in the central nervous system [29]. An animal’s behavioural repertoire is influenced by the existing neural architecture and its physiology, which can be strongly affected by changes in regulatory regions and translational repressive mechanisms. Such repressive factors are also found to be relevant during memory formation [30] and can include translation initiation or elongation factors [31, 32] and microRNAs [33]. When studying complex behavioural traits like learning ability and memory retention, it is therefore insightful to consider both gene expression differences and changes in gene allele frequency in parallel. ‘Evolve-and-resequence’ experiments are a powerful way of detecting allele frequency changes in response to selection on various traits [34–38]. Here we use this approach to screen both the genome and the transcriptome of a parasitoid wasp for loci that respond to selection on learning ability.

The parasitoid wasp *Nasonia vitripennis* has become a model for evolutionary genetics approaches since it combines convenient genetic features with an interesting biology and behavioural repertoire [39]. The females are very sensitive to cues that help them find concealed hosts in a complex environment, making these wasps excellent subjects for studying associative learning ability. QTLs for memory-related phenotypes were identified by introgressing two related *Nasonia* species [40]. Also, differences in gene expression induced by a learning experience were found between the same two species, resulting in a list of candidate genes that may regulate long-term memory formation [40]. These studies benefit from using isofemale lines with limited genetic variation, which increases the contrast of a potential genetic difference between the different lines. However, such studies do not reflect situations under natural conditions where polygenic adaptation, especially at the early stages of selection, draws on standing genetic variation in populations [41, 42].

In this study, we exploit variation segregating in a natural population to identify the genetic and transcriptional basis of associative learning ability. Specifically, we assessed how selection on associative learning and memory formation impacts gene allele frequency and gene expression. In an earlier study, replicate lines of *N. vitripennis* were selected for rapid associative learning ability in associating a colour with host presence [8]. Associative learning ability responded within 10 generations of selection and proved to also extend to associative learning of other cues and rewards besides the visual associative learning task that was selected for [8]. We jointly analysed genome-wide allele frequencies and gene expression levels in head tissue of adults of the replicated, evolved populations. If genes involved in learning ability are under *cis*-regulation, we might expect to find significantly diverged SNPs in the regulatory regions of genes that show differential expression. Alternatively, we may expect that sequence evolution and regulatory changes do not involve the same genomic regions, but affect genes that are functionally related or part of the same interaction network. We therefore constructed a gene network using the genes identified by our genomic and transcriptomic analyses. This integrative approach reveals how a complex trait like associative learning ability can readily respond to selection via a complex interplay between regulatory and sequence evolution.

## Results

### Selection for associative learning ability

**Fig. 1:**
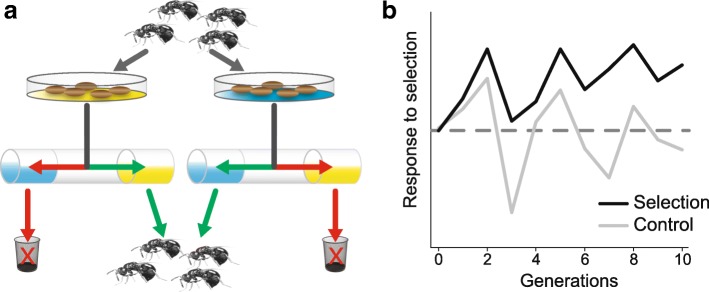
Artificial selection on associative learning ability in *N. vitripennis.***a** Schematic overview of the selection experiment. Females were allowed to oviposit on host pupae placed on either a yellow or blue background. 24 h later, the females were placed in a transparent tube with yellow and blue outer ends. Only females that chose the background colour corresponding to the colour on which they had been trained were used to found the next generation. This procedure was repeated ten generations in four replicate selected lines. Each selected line was paired to a control line that was treated following the same procedure, except that in the control lines, females were randomly chosen from the tested wasps. Nasonia’s after a photo by Jitte Groothuis. **b** Changes in associative learning ability (mean performance index over four replicate lines) over ten generations of selection in selected and control lines. For details, see [8]

Experimental procedures and phenotypic responses to selection for each pair of selected and control lines are summarized in Fig 1. Associative learning ability responded swiftly to selection: within 10 generations of selection, the response in forming learned associations was significantly increased in the four selected lines [8]. The baseline associative learning ability of the four control lines showed no consistent in- or decrease.

### Genome sequencing statistics

To identify the genetic variants contributing to evolved differences in associative learning ability, we performed pooled whole-genome sequencing on four selected and four control *N. vitripennis* lines. We obtained on average 77,796,493 reads per library, totaling 622,371,946 genomic read pairs across the eight samples. The average mapping rate to the reference genome was 92%. Mean coverage per sample ranged between 25x and 139x. Sequencing statistics for the eight genomic DNA libraries are summarized in Additional file 1.

### Allelic divergence

**Fig. 2:**
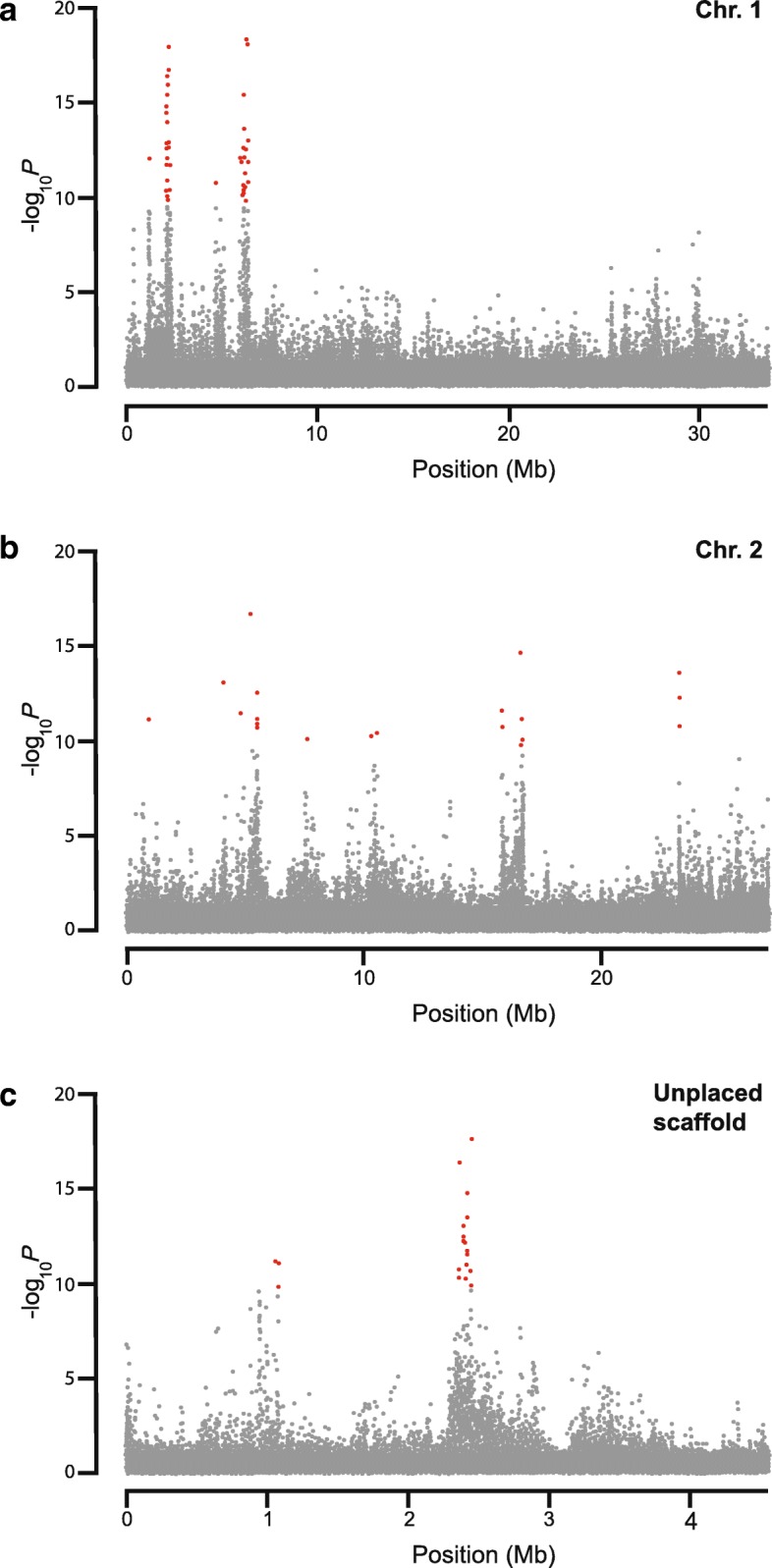
Genome-wide association study of associative learning ability. Manhattan plots for selection treatment in the full dataset (including all four replicate pairs). The –log_10_*P* values are plotted against the position on each genomic scaffold. Red dots indicate variants for which the FDR-adjusted *P* < 5e-6. **a** Chromosome 1 (scaffold NC_015867.2) (**b**) Chromosome 2 (scaffold NC_015869.2) (**c**) Unplaced scaffold NW_001820749.1

A total of 2.6 M variant positions passed a series of stringent filters (see Methods). Patterns of allelic divergence differed between pairs of selected and control lines (Additional file 2). Most of these allele frequency differences are likely to be due to genetic drift in each of the lines. Only variants that differed consistently between the four selected and four control lines are likely to be due to the selection regime. While there may also have been line-specific responses to selection, such effects would be indistinguishable from genetic drift. We therefore used a generalized linear mixed model to identify variants that had consistently diverged in response to selection for associative learning ability, while taking into account differences in sequencing depth and idiosyncratic differences in allele frequency between replicates [35]. After adjusting for multiple testing, we identified 118 variants as significantly diverged between selected and control lines (Additional file 3). These variants were distributed over 12 genomic scaffolds, with 1 to 46 significant variants per scaffold and clustered in 51 distinct genomic regions separated by 60 kb or more. Three scaffolds contained 19 or more significant variants (Fig. 2). *F*_ST_ values were elevated in regions containing clusters of consistently diverged SNPs (Additional file 2).

### Effect prediction

We determined which genes were potentially affected by the 118 consistently diverged SNPs by selecting all genes within 10 kb of each of these SNPs according to the official annotation of the *N. vitripennis* genome. In addition to exons and introns, we included the regions 10 kb up- and downstream of each gene, as these may include important regulatory sites. A total of 128 genes were located within 10 kb of a consistently diverged SNP (Additional file 4). We retrieved gene ontology (GO) terms associated with each gene. If our selection regime primarily affected genes with cognitive functions, GO terms associated with such function would be overrepresented among our gene set. No GO terms were overrepresented among genes associated with consistently diverged SNPs, indicating that a substantial number of consistently diverged variants had likely diverged as a result of hitchhiking, due to linkage to loci under selection.

**Fig. 3:**
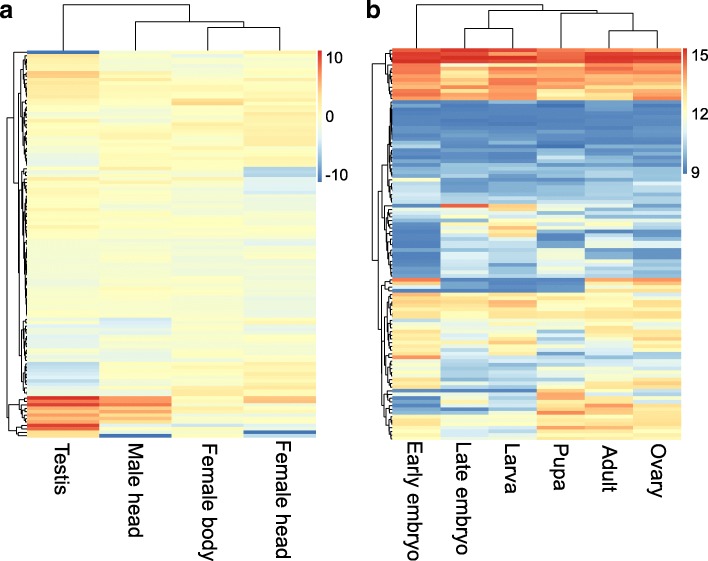
Tissue- and life stage-specific expression of candidate genes. Heatmap of expression patterns of the candidate genes identified in the genomic analysis in different (**a**) tissues (log transformed) and (**b**) life stages. Source: WaspAtlas

Similarly, we did not expect the 128 genes of interest to show an overall bias towards expression in any particular tissue because of hitchhiking. However, we expected that genes involved in associative learning ability would be most strongly expressed in head (brain) tissue. We used data available in WaspAtlas [43] to assess in which tissues these 128 genes are known to be (primarily) expressed, comparing relative expression values in the available tissue-specific expression data (female and male head, female whole-body and male testis). The results (Fig. 3a) showed that 30 of the 128 genes in the vicinity of diverged SNPs had increased expression in female head tissue. The genes with the most strongly increased expression in female heads included: LCCH3, serine protease 133, putative polypeptide N-acetylgalactosaminyltransferase 9, lachesin, prickle-like protein 3 and prominin-1. By contrast, a small group of genes were conspicuously higher expressed in testis tissue (Fig. 3a). We expect that chance associations are the cause of this pattern, due to hitchhiking of many significantly diverged genes with a small number of brain-related genes that responded to selection. Alternatively, by selecting only females during the selection experiment, we may have skewed allele frequency of sexually antagonistic genes.

Another possible explanation for the paucity of genes with relatively high expression in female head tissue among genes near diverged SNPs is that important genes involved in brain development may act in earlier developmental stages, in particular in the pupal stage. To investigate this, we compared the levels of expression of genes near significantly diverged SNPs in ovaries (eggs), early and late embryos, larva, pupae and adults using data available in WaspAtlas. The results (Fig. 3b) show clusters of genes that are either highly or lowly expressed during a particular developmental stage across all tissues. About a third of the candidate genes show stage-specific expression (Fig. 3b). Genes showing highest expression in pupae, but low expression in embryonic, larval and adult stages included low-density lipoprotein receptor-related protein 6-like, putative odorant binding protein 62, and two uncharacterized proteins.

Most diverged SNPs (62.7%) were located in introns. Smaller numbers of diverged SNPs were located outside genes (28.8%) or in coding regions (8.5%). For eight genes we found significantly diverged SNPs in their coding regions (Table 1, Additional file 5). Four SNPs resulted in amino acid changes in genes encoding pyrokinin, turripeptide Pal9.2like, kinectin and an uncharacterized protein (Table 1). A further five SNPs in coding regions did not change the amino acid sequence (Table 1).

**Table 1:** Genes carrying consistently diverged SNPs in their protein-coding regions

gene name	gene accession	mutation type
anaphase-promoting complex subunit 1	LOC100122916	synonymous
cytoplasmic dynein 2 light intermediate chain 1	LOC100121423	synonymous
isoleucyl-tRNA synthetase 2	Iars2	synonymous
kinectin-like	LOC100680081	missense
pyrokinin/capa receptor 2	LOC100120473	missense
tctex1 domain-containing protein B (aka TctexB)	LOC100463506	missense
turripeptide Pal9.2-like	LOC100680056	missense
turripeptide Pal9.2-like	LOC100680056	synonymous
clarin-3	LOC100122852	synonymous

### Transcriptome sequencing statistics

To compare gene-specific expression levels between the eight replicate *N. vitripennis* lines (four selected and four control lines), we sequenced mRNA extracted from female heads. We obtained on average 101 × 10^6^ raw PE 100 bp reads per library of which we retained 95% high quality reads on average. Removing transcripts with no or low expression yielded a total of 17,007 expressed transcripts, out of a total of 26,079 in the reference transcriptome. A complete overview of the sequencing information is presented in Additional file 6.

### Gene expression

**Fig. 4:**
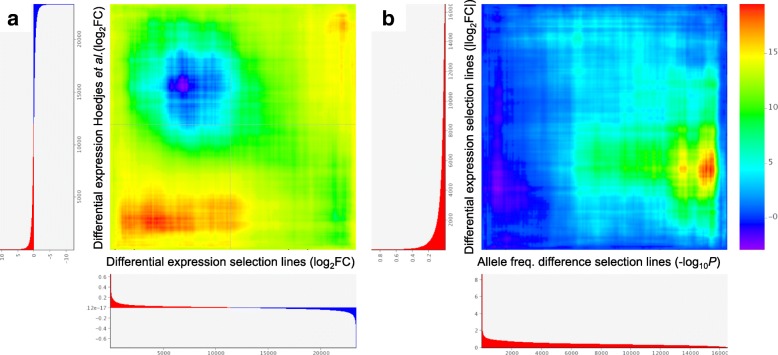
Rank-rank hypergeometric overlap (RRHO) analysis, comparing the differential gene expression results to (**a**) results obtained by [40], who compared gene expression between trained and untrained wasps, and (**b**) allele frequency difference (ranked by *P*). Overrepresentation of transcripts in the lower left and upper right of (**a**) indicate correspondence between the two datasets at the top and bottom of the sorted gene expression lists. No such overlap is seen in (**b**), where the only overrepresentation occurs at low *F*_ST_ and non-differential gene expression

We used three complementary analyses to identify transcripts that showed significant and large changes in expression, consistent across the four pairs of selected and control lines. Differential expression analysis in edgeR yielded 21 significant genes, pairwise χ^2^ tests yielded 23 significant genes and principal component analysis yielded 17 significant genes (see Methods for details, Additional files 7, 8, 9 and 10). Combining these sets of transcripts and removing those with less than two-fold difference in expression between control and selected lines resulted in 36 significant genes (Additional file 10). Rank-rank hypergeometric overlap (RRHO) analysis showed correspondence between our differential expression results and those obtained by [40], who compared gene expression in trained and untrained *N. vitripennis* (Fig. 4a)*.* Specifically, there was overrepresentation of transcripts that were either up- or downregulated in both our selected lines and in trained wasps (Fig. 4a). Surprisingly, none of these transcripts matched genes identified as having diverged in allele frequency between selected and control lines in the genomic analysis. RRHO analysis similarly showed no overlap between the lists of differentially expressed genes and those showing differences in allele frequency (Fig. 4b). However, when we examined pairs of selected and control lines separately, we did observe weak, but significant correlations between differential gene expression and *F*_ST_ for three of the four pairs (Additional file 11).

**Fig. 5:**
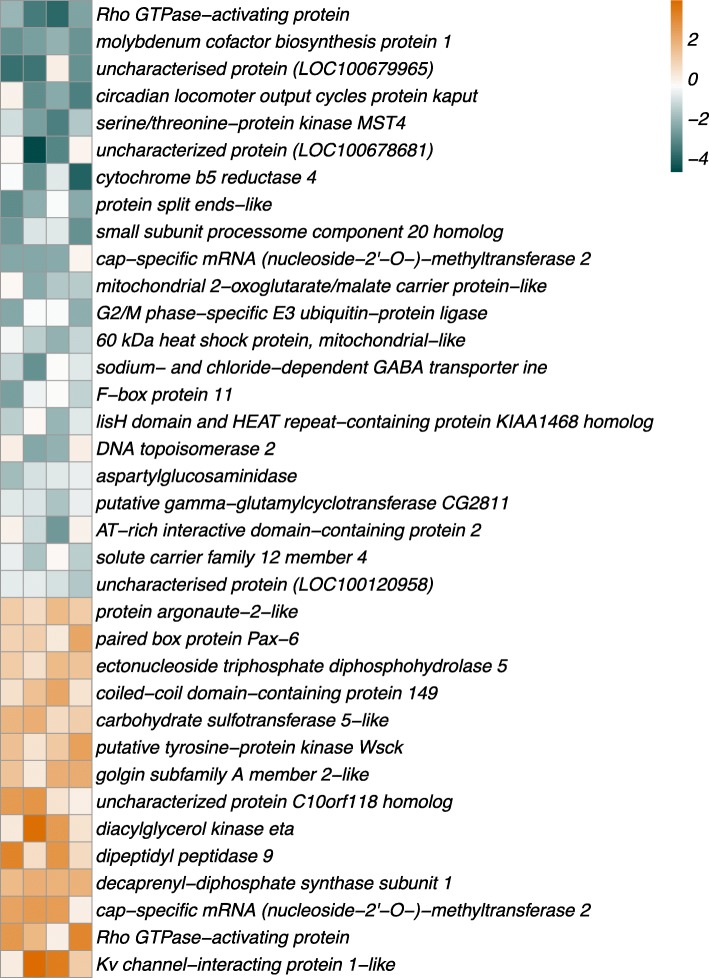
Evolved expression changes in response to selection for increased associative learning ability. Heatmap shows log_2_ Fold Change between control and selected lines for four replicated pairs of lines (in columns), and 36 significant transcripts in rows (sorted from low to high Fold Change). Positive Fold Change values (in green) represent increased expression in selected compared to control lines, and negative values (in red) represent reduced expression. See Table 2 and Additional file 10for details and statistics

For 14 transcripts, expression was elevated in the selected lines while the remaining 22 transcripts showed reduced expression (Fig. 5, Table 2). Neither of these sets of transcripts was enriched for any GO term. Interestingly, seven transcripts showed completely eliminated expression as a result of selection in at least three of the four replicates, while for seven other transcripts the reverse was the case: from zero expression in the control lines to high expression in the selected lines (Additional file 12). Thus, for 14 out of 36 significant transcripts, the evolved increase in associative learning ability was associated with a near-complete on or off switching of expression. Finally, two genes each expressed two alternative splice variants that showed complete opposite patterns of expression in response to selection, i.e. one transcript went from low to high expression upon selection while the other transcript of the same gene went from high to low expression. The first gene, Rho GTPase-activating protein, showed an 8-fold reduction in expression of one of the splice variants, the greatest among all transcripts, while the other transcript had virtually zero expression in the control lines but increased to the expression level of the alternative splice variant upon selection. The other gene, cap-specific mRNA (nucleoside-2’-O-)-methyltransferase 2, displayed a similar pattern (Additional file 13).

**Table 2:** Evolved expression changes in response to selection for enhanced associative learning ability. Thirty-six transcripts, expressed from 34 loci, showed evidence of evolved expression regulation that was consistent across four replicate pairs of lines, as identified by three complementary methods (see Methods and Additional file 8). Transcripts are in order of increasing Fold Change, with high Fold Change indicating elevated expression in selected lines

gene name	gene accession	transcript accession	log2 Fold Change	edgeR P_adj_	pairwise tests P_adj_	PCA correlation ρ_pearson_	P
Rho GTPase-activating protein^b^	LOC100118685^b^	XM_008205242.1^b^	-3	0	0	0.8	0
molybdenum cofactor biosynthesis protein 1	LOC100118555	XM_008212104.1	−2.6	0	0	0.7	0.02
uncharacterised protein (LOC100679965)	LOC100679965	XM_008216564.1	−2.6	0	1	0.3	0.18
circadian locomoter output cycles protein kaput	LOC100114103	XM_008216217.1	−2.2	0	0.09	0.5	0.06
serine/threonine-protein kinase MST4	LOC100121999	XM_008213253.1	−2.2	0	0	0.7	0.01
uncharacterized protein (LOC100678681)	LOC100678681	XM_003425395.2	−2.1	0.83	0.43	0.6	0.02
cytochrome b5 reductase 4	LOC100117767	XM_008209788.1	−2.1	0.74	0	0.2	0.27
protein split ends-like	LOC100123328	XM_008214414.1	−2	0	0	0.2	0.22
small subunit processome component 20 homolog	LOC100122027	XM_008212760.1	−1.9	0	0	0.3	0.16
specific mRNA (nucleoside-2’-O-)-methyltransferase 2^a^	LOC103315388^a^	XM_008203871.1^a^	− 1.8	0.01	0.6	0.6	0.02
mitochondrial 2-oxoglutarate/malate carrier protein-like	LOC100121662	XM_008219522.1	−1.4	0.06	0.02	0.6	0.02
G2/M phase-specific E3 ubiquitin-protein ligase	LOC100115982	XM_008205146.1	−1.4	0.99	0.01	0.2	0.3
60 kDa heat shock protein, mitochondrial-like	LOC100114031	XM_001599995.3	−1.4	0.02	0	0.7	0.01
sodium- and chloride-dependent GABA transporter ine	LOC100115303	XM_008210619.1	−1.4	0.4	0.01	0.2	0.31
F-box protein 11	LOC100115478	XR_512626.1	−1.2	1	0	0.1	0.4
lisH domain and HEAT repeat-containing protein KIAA1468 homolog	LOC100120301	XM_008213739.1	−1.2	0.94	0.09	0.7	0.01
DNA topoisomerase 2	LOC100117297	XM_008208620.1	−1.1	1	1	0.7	0.01
aspartylglucosaminidase	LOC100119424	XM_008213772.1	−1.1	0.43	0	0.1	0.39
putative gamma-glutamylcyclotransferase CG2811	LOC100122076	XM_008213266.1	−1	0.11	0	0.7	0.01
AT-rich interactive domain-containing protein 2	LOC100121989	XM_008218909.1	−1	1	1	0.7	0.01
solute carrier family 12 member 4	LOC100116848	XM_008210249.1	−1	0.53	0.04	0.2	0.21
uncharacterised protein (LOC100120958)	LOC100120958	XM_008211884.1	−1	0.55	0	0.5	0.05
protein argonaute-2-like	LOC100123519	XM_008216662.1	1.1	0.03	0	0.3	0.15
paired box protein Pax-6	LOC100118963	XM_001602773.3	1.1	0.05	0.02	0.1	0.51
ectonucleoside triphosphate diphosphohydrolase 5	LOC100678150	XM_008211788.1	1.1	0.04	0	0.6	0.03
coiled-coil domain-containing protein 149	LOC100122426	XM_008206132.1	1.1	1	0.01	0.7	0.02
carbohydrate sulfotransferase 5-like	LOC100116691	XM_001601071.3	1.3	0.02	0	0.3	0.17
putative tyrosine-protein kinase Wsck	LOC100677925	XM_008204866.1	1.3	0.28	0	0.2	0.21
golgin subfamily A member 2-like	LOC100116846	XM_008209761.1	1.4	0.03	0.42	0.5	0.04
uncharacterized protein C10orf118 homolog	LOC100121426	XM_008204491.1	1.4	0	1	0.2	0.29
diacylglycerol kinase eta	LOC100120935	XM_008212357.1	1.7	0.03	0.38	0.5	0.06
dipeptidyl peptidase 9	LOC100123733	XM_008209082.1	1.8	0.02	0	0.3	0.15
decaprenyl-diphosphate synthase subunit 1	LOC100118369	XM_008211982.1	1.8	0	0	0.8	0.01
cap-specific mRNA (nucleoside-2’-O-)-methyltransferase 2^a^	LOC103315388^a^	XM_008203872.1^a^	1.8	0	1	0.7	0.01
Rho GTPase-activating protein^b^	LOC100118685^b^	XM_001602542.3^b^	1.8	0.01	1	0.1	0.5
Kv channel-interacting protein 1-like	LOC100121497	XM_008205258.1	2.1	0	0.14	0.5	0.05

^a^Two transcripts for this locus show opposite patterns of evolved expression changes

^b^Two transcripts for this locus show opposite patterns of evolved expression changes

### Gene network analysis

**Fig. 6:**
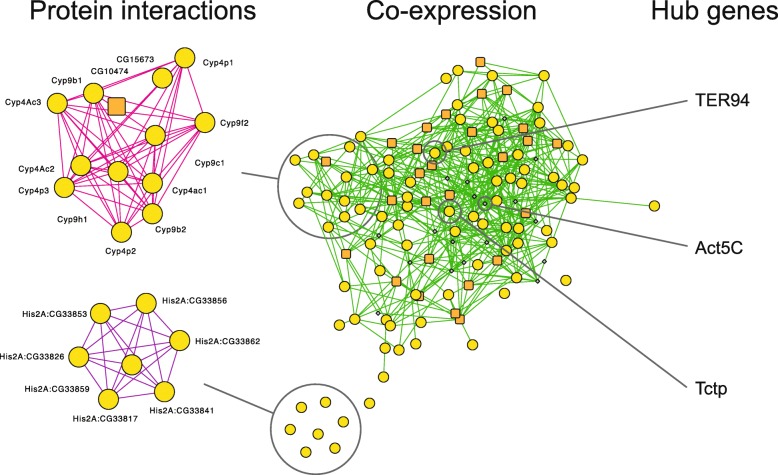
Gene network for *D. melanogaster* orthologs of genes that responded to selection for increased associative learning ability in *N. vitripennis.* Nodes represent genes and edges are based on co-expression (center). Two subnetworks where edges represent shared protein domains are shown on the left. Three putative hub genes that each have physical interactions with > 6 other genes within the network are indicated on the right. Genes with significantly diverged SNPs are indicated in yellow circles, differentially expressed genes in orange squares and highly connected genes added by GeneMania in grey

To explore potential functional relationships between the candidate genes identified by our genomic and transcriptomic analyses, we constructed a gene network for all 113 *Drosophila melanogaster* orthologs of our candidate genes (Additional files 4 and 6) in GeneMania [44]. This resulted in a co-expression network that included 77 genes with diverged SNPs and all 27 differentially expressed genes for which a *Drosophila* ortholog was known (Fig. 6a). The co-expression network consisted of 638 edges and contained two sub-networks of genes with shared protein domains (13 and 7 genes per clusters; Fig. 6b). Furthermore, several of the genes in the network code for proteins that have been shown to physically interact with the proteins of several other genes in the network. These genes may constitute putative hub genes in the network and included ATPase TER94.

## Discussion

We identified 118 genomic loci that showed consistent differences in allele frequency between replicated selected and control lines. A total of 128 genes were located in close proximity to significantly diverged loci. Transcriptome analysis of the same selected and control lines identified 36 transcripts as consistently differentially expressed. None of these matched the significantly diverged genomic loci. However, network analysis showed that many of the genes near diverged loci clustered with the differentially expressed genes in a single co-expression network.

Four of the significantly diverged SNPs at the 118 candidate genomic loci changed the amino acid sequence of the genes product. These SNPs resulted in missense mutations in the genes pyrokinin (involved in neuropeptide signaling pathway), turripeptide Pal9.2like (an ion channel inhibitor and neurotoxin), kinectin (transmembrane protein interacting with RhoGTPases) and an uncharacterized protein. A further five diverged SNPs in coding regions did not change the amino acid sequence. This included a synonymous SNP in the gene clarin− 3, which encodes a transmembrane protein expressed in cochlear hair cells and neural retina and which is associated with cognitive performance in Parkinson’s disease in human GWAS [45]. The remaining SNPs were located in introns or outside protein-coding regions. Some of these were in or near genes with known functions in insect learning (see [46] for an overview) and may have affected their expression. These included genes encoding serine protein kinases, gated channels, cytoskeleton components (actin), a splicing factor, several neuropeptides and several odorant-binding proteins. In addition, candidate SNPs were found near several histone-related genes and at least 1 methyltransferase, pointing to epigenetic regulation. One SNP was located downstream of an ortholog of *latheo*, of which knock-down mutants in *D. melanogaster* show learning and memory deficits [47].

The 36 differentially expressed genes likewise included genes encoding a serine protease, a transporter gene, a kv channel and a cap-specific methyltransferase. Especially interesting is a differentially expressed gene encoding a RhoGTPase, as one of the genes carrying a missense candidate SNP identified in the genomic analysis encoded a kinectin, which is a transmembrane protein that interacts with RhoGTPases. RhoGTPases are key regulators of dendritic morphology that integrate environmental cues, such as neuronal activity [48].

None of the differentially expressed genes matched those identified in the genomic analysis, although differential expression and allelic divergence were weakly correlated at the level of pairs of selected and control lines. A number of factors may account for this observation. First, some of the diverged genes may function in early development, rather than in adults, which was the only life stage we sampled for the transcriptome analysis. Several diverged genes did indeed show strongest expression during the pupal stage (WaspAtlas). This included low-density lipoprotein receptor-related protein 6-like, the mammalian ortholog of which is critical for synaptic function and cognition [49]. In humans, variants in this gene have been linked to Alzheimer’s disease risk [49]. Second, some of the diverged loci may affect protein structure and function, rather than transcription. Last, the candidate genes showing evolved expression differences may all be regulated in more complex, indirect ways. Gene network analysis revealed all differentially expressed genes to be co-expressed with 77 of the genes with diverged SNPs, at least in *Drosophila.* Furthermore, three genes were known to interact physically with many other genes in the network. This included the ATPase TER94, which is involved in dendrite morphogenesis and neuron apoptosis in *D. melanogaster* [50]*.* Further work is required to distinguish between these possibilities.

Evolved differences in gene expression associated with improved associative learning, as studied here, at least partially targeted existing regulatory pathways of the process of learning and formation of memory per se. The list of most strongly up- or downregulated genes showed overlap with that obtained by an earlier study [40], that compared the head transcriptomes of *N. vitripennis* with and without a learning experience. This suggests that the evolutionary response to selection in terms of gene expression attenuated the gene expression differences induced by learning in unselected wasps. This effect was only detected using sensitive RRHO analysis, but not by comparing only the lists of differentially expressed genes. Similarly, [46] identified genes that were differentially expressed between conditioned and unconditioned individuals of two other species of parasitoid wasp and found that even for different types of memory formation (e.g. short- versus long-term memory formation) within one species, the overlap in differentially expressed genes was only 9% [46]. The diffuseness of overlap between transcriptomic studies of different aspects of learning ability points to the highly specific nature of gene expression involved in different processes of learning and memory.

## Conclusions

In conclusion, both the genomes and the transcriptomes of the selected lines changed in response to selection on associative learning ability, however there was no overlap between the two gene sets. Further studies are required to assess the generality of this finding. A recent study on the adaptive response to toxicity in populations of killifish found genomic and transcriptomic changes to affect the same pathways [51]. On the other hand, artificial selection on resistance against parasitoid attack in *D. melanogaster* yielded allelic changes at many loci [34], but no differential expression in any of these loci after parasitoid attack [52], and only very limited overlap with genes that changed in expression during egg-larval development in the selected lines [53]. In our study, we found many of the diverged gene loci to cluster in the expression network with the differentially expressed genes. We therefore conclude that selection acted on loci that affected the phenotype through complex mechanisms, including gene interactions and gene regulation at multiple loci.

## Methods

### Study organism & selection experiment

*Nasonia vitripennis* (Hymenoptera: Chalcidoidea) is a generalist gregarious wasp that parasitizes pupae of large dipterans (e.g. Calliphoridae) [54]. The founding *N. vitripennis* population for our selected and control lines was the HVRx strain which was originally established from females collected at five field sites in the Netherlands and is maintained with relatively high genetic variance [55]. Analysis of 40 microsatellite loci showed that the effective population size is kept at *N*_e_ = 173, expected heterozygosity was He = 0.56 and allelic richness was around 3.4 [55]. Genomic SNP density per 100 kb window averaged 0.13% [55]. At the start of the selection experiment females were randomly assigned to four pairs of selected and control lines (A, B, C, and D). Environmental factors like season, air pressure and (seasonal) host quality can drastically influence behaviour and motivation, introducing substantial variation in the learning response between generations. To control for this, sets of selected and control lines were always conditioned and tested on the same day [8]. We selected for rapid associative learning and memory retention in female wasps using appetitive conditioning to associate a colour with a host reward. Each generation, the females and males that emerged were kept together for 2–3 days for mating and maturation, after which ca. 160 mated females per line were conditioned (Fig. 1a). To avoid increasing sensitivity to a particular colour, half of the wasps were conditioned on the colour blue and the other half on the colour yellow at each generation. When given a choice between the reinforced colour and a neutral colour in a T-maze 24 h later, wasps that learned the association between colour and reward will move towards the conditioned colour. As a measure of this learned preference we calculate a ‘performance index’ based on the distribution of wasps in the T-maze between the two reciprocal groups (conditioned on yellow or blue). This index can be expressed as a percentage and ranges from 0 (no learned preference) to 100 (perfect learned preference) and controls for innate colour preferences [8]. Only the wasps that demonstrated a learned preference for the conditioned colour were chosen for the selected lines (Fig. 1a), while in the control lines wasps were chosen randomly. Each generation, 50 mated females per line were allowed to reproduce. The experiment was continued for 10 generations under continuous selection, the results and details of this experiment are described in [8]. Although learned preferences showed considerable variation between lines and generations, the mean learning response (performance index) of the selected lines increased over time, while that of the control lines did not (Fig. 1b; [8]). No detrimental effects of the selection regime were observed; we detected no difference in relative brain volume, lipid content or body size between selected and control lines and a minor difference in longevity. Innate colour preference of the wasps remained constant throughout the experiment [8].

### DNA extraction, sequencing and alignment

Pools of 40 females per selected and control line were collected 24 h after being conditioned on a colour, as during the selection experiment. DNA was extracted from these pools using the following protocol. Groups of ten females were rinsed in 70% ethanol, vacuum dried and crushed in 200 μl phosphate buffered saline. After adding 200 μl Nuclei Lysis Solution, 5 μl RNAse (4 mg/ml, Promega®) and 4 μl Proteinase K (20 mg/μl, Roche®), the samples were incubated for 15 min at 60 °C. A further 340 μl DNA Lysis buffer (Promega®) was added and after a spin down (10 min. at full speed) the supernatant was collected on a spin column (Promega®). After 3 wash steps the DNA was eluted in 100 μl H_2_O.

Length and integrity of the DNA molecules were checked on a 1% agarose gel. Concentrations of DNA and RNA were measured on a Qubit® device and purity was assessed using Nanodrop. Equal amounts from the four DNA isolations per line were pooled, creating eight DNA samples for sequencing.

Each pooled DNA sample was fragmented to 300 bp using a Covaris sonicator. DNA fragments were size selected and Illumina libraries were constructed at the Leiden Genome Technology Center at the Leiden University Medical Center (Leiden, The Netherlands). All samples were sequenced on an Illumina HiSeq2000 (100 bp paired-end). Additional sequence data was generated for one sample (selected line D) on an Illumina NextSeq500 (125 bp paired-end). Initial quality checks of the raw reads were conducted using SGA preprocess [56] and fastqc [57]. Reads were mapped to the *N. vitripennis* reference genome (nvit_2.1) using Bowtie2 [58] using default parameters. Duplicate reads were removed using PicardTools (https://broadinstitute.github.io/picard/) and indel realignment was conducted using GATK [59].

### Genotyping

A single mpileup file was drawn from the bam files for the eight selected and control lines using Samtools [60]. This was converted to a sync file as input for Popoolation2 [61] using the script mpileup2sync.jar with –min-qual 20. The sync file was then converted to a vcf file using a custom python script. A total of 72 M variant positions were initially identified as biallelic. We selected only variant positions for which the total coverage was > 10 for all selected and control lines and the nonreference allele had a coverage > 5 for at least one of the lines. An additional 11 M variant positions were identified as triallelic. Most (89.1%) of these loci were actually biallelic, with either both alleles different from the reference or a third ‘allele’ with very low coverage that was probably a sequencing error. We selected loci that had an overall coverage > 10 for all selected and control lines and for which two (but not three) alleles had a coverage > 5 in at least one of the selection lines. A total of 2.6 M variant positions passed these criteria.

### *F*_ST_

To visualize genetic divergence between each selected line and its paired control line, we calculated *F*_ST_ in 10 kb windows using Popoolation2. The original sync file (see under genotyping) was used and the maximum coverage was set at 2%.

### Generalized linear mixed model

To identify consistent allele frequency differences between selected and control lines while controlling for the extreme variance in sequencing depth among the lines, we implemented a GLMM using the R package lme4 following [35]. Read counts for the two alleles at each variant position were the response variable in the model and errors were assumed to follow a binomial distribution. Selection regime (selected / control) was specified as a fixed effect and line (one of eight; selected A, control A, etc.) as a random factor. *P* values for each variant position were obtained using Wald tests and were FDR-adjusted for multiple testing using the method of [62]. We considered any variant with an adjusted *P* value below 5e-6 statistically significant. The potential effects on nearby genes were annotated using SnpEff [63]. GO enrichment was assessed using the overrepresentation module in WaspAtlas [43]. The level of expression of each gene during different developmental stages and tissues was investigated using data available in WaspAtlas. Five significantly diverged SNPs in coding regions were validated using Sanger sequencing. The same pooled DNA samples that were used for Illumina sequencing were used as templates. Allele frequencies of the focal SNPs were estimated from the height of each of the two overlapping peaks in Vector NTI v.11.0.

### RNA extraction, sequencing and alignment

For each of the four selected and four control lines, we dissected heads from 15 individual, 4-day old, adult females that had been collected 24 h after being conditioned on a colour. All heads were snap frozen in liquid nitrogen and stored at − 80 °C until RNA isolation, pooling all heads from the same line into a single sample. RNA was extracted using the RNeasy® Micro kit of Qiagen®, following the manufacturer’s protocol “Purification of Total RNA from Animal and Human Tissues” and eluted in 2 times 14 μl H_2_O. RNA concentration and purity was assessed using Qubit. mRNA size selection, reverse transcription, paired-end library preparation and sequencing were performed in-house at Macrogen. The eight paired-end libraries of 2 × 100 bp (average insert size 186 bp) were sequenced across three lanes on an Illumina HiSeq 2500. Read quality using FastQC v. 0.10.1 (http://www.bioinformatics.babraham.ac.uk/projects/fastqc/). Trimmomatic v. 0.3 [64] was used to trim and remove low quality reads. We removed reads containing adaptor sequence, and removed bases if the Phred score over a four base sliding window was below 15, or if the leading or trailing bases had a score below 3. We dropped read pairs entirely if either read pair after trimming was less than 36 bases. We used RSEM [65] 1.2.26 with Bowtie2 [58] v. 2.2.6, both with standard parameters to map the reads to the reference transcriptome (GCF_000002325.3_Nvit_2.1_rna.fna from NCBI, annotation version 101) and quantify expression (i.e. read abundance) at transcript level. This version of the reference transcriptome was constructed with the aid of RNAseq data from several different tissues, including adult heads [66]. Next, we filtered the resulting count data by minimum expression, only retaining transcripts whose expression was at least one Count Per Million (CPM) in at least one of the eight libraries.

### Differential expression analysis

We aimed to identify transcripts showing expression responses to the selective environment that were concordant across the four replicate line pairs. Given the strong line-by-selection interactions (see principal component analysis below), which with our experimental design could not be estimated directly, we employed three complementary analysis methods. To reduce the number of false positives, we combined these methods with strict filtering criteria. First, we used standard general linear model differential expression analysis, as implemented in edgeR to model untransformed raw count data for each transcript separately as a function of selection treatment and line, but not their interaction, given that our design did not allow for the independent estimation of the line-by-selection interaction. We tested the effect of the selective environment by performing likelihood ratio tests between models with and without the selection treatment effect, yielding a likelihood ratio and corresponding *P* value, which we corrected for multiple comparisons using Benjamini and Hochberg’s False Discovery Rate [62] (FDR), accepting an FDR of 0.05. We only retained transcripts that showed concordant expression changes across all four line pairs, and had an absolute Fold Change > 2, resulted in 21 significant genes. Second, we tested independently evolved expression differences within each line pair using a series of pairwise χ^2^ tests to compare untransformed, raw counts between the selected and control library of each line pair (each a pool of 15 individuals). The subtotals across all genes for the selected and control library were used to compute expected counts (under the null hypothesis of no differential expression). This yielded a χ^2^ and corresponding P value for each transcript, and we retained only transcripts with FDR < 0.05 in all four comparisons, concordant expression changes in all four pairs, and an absolute Fold Change > 2. This resulted in 23 significant transcripts, eight of which were unique to this method. These eight genes were missed by edgeR, possibly due to confounding interaction effects. Finally, to explore the variance structure in the expression data, we performed a principal components analysis (PCA) using the function prcomp in R [67] (v.3.3.2) on the expression data, first normalised to CPM using Trimmed Methods of Mean (TMM) as implemented in edgeR [68] v. 3.14.0, and log transformed. This analysis revealed strong effects of selection line on transcriptional variation, both independent of, and in interaction with the selective regime. PC 1 separated line pairs A and B from C and D, independent of selective regime, and PC 2, 4, and 5 separated selected from control lines for some replicates, but not for all four. Only PC 6 consistently separated selected from control lines in all four replicates, which was the pattern we were most interested in (Additional file 7). We then used this PC to find consistently evolved expression changes. For each gene, we computed a Pearson’s correlation between its normalized expression and the loading of that gene along PC 6. Given our low power to detect significant correlations with only eight data points, we did not correct the resulting *P* values for multiple comparisons, but instead kept the transcripts with *P* < 0.05. We filtered again by concordance in expression across the four replicate line pairs, as well as by Fold Change > 2. This final method yielded 17 significant transcripts, four of which were unique to this method. See Additional files 7,8, 9 and 10 for an overview of the significant transcripts discovered by each method.

We extracted transcript names, gene names and genomic location for each transcript using its accession in the reference transcriptome, and the reference gff3 annotation file at NCBI, both for Nvit2.1, annotation 101 (https://www.ncbi.nlm.nih.gov/assembly/GCF_000002325.3/), supplemented with additional annotation information from WaspAtlas [43]. We used rank-rank-hypergeometric overlap analysis [69], as implemented on http://systems.crump.ucla.edu/rankrank/rankranksimple.php, to compare the degree of differential gene expression between selected and control lines to differential gene expression obtained by [40] and allele frequency differences. This algorithm steps through two gene lists ranked by either the degree of differential expression or allele frequency difference (*P* from GLMM), successively measuring the statistical significance of the number of overlapping genes [69]. To further assess the correspondence between the differential expression and allele frequency differences, we compared these values for each pair of selected and control lines using Pearson’s χ^2^ tests.

## Additional files

Additional file 1: **Table S1.** Sequencing information for each of the eight pooled genomic DNA libraries. (XLSX 9 kb)

Additional file 2: **Figure S1.** Manhattan plots of *F*_ST_ values for each of the four pairs of selected versus control lines (A, B, C and D). Areas highlighted in red correspond to the clusters of significant SNPs in Fig. 1. (a) Chromosome 1 (scaffold NC_015867.2) (b) Chromosome 2 (scaffold NC_015869.2) (c) unplaced scaffold NW_001820749.1 (note difference in y-axis scaling). (PDF 19568 kb)

Additional file 3: **Table S2.** Allele frequencies of the 118 significantly diverged SNPs in the selection and control lines. (XLSX 19 kb)

Additional file 4: **Table S3.** Genes underlying consistently diverged SNPs. Included are genes carrying candidate SNPs in their gene bodies or in the regions 10 kb up- or downstream. (XLSX 93 kb)

Additional file 5: **Figure S2.** Sanger sequencing validation of five significantly diverged SNPs in coding regions (see Table 1). Frequency of the alternative allele (missense or synonymous) was estimated from the peak height in Sanger sequencing trace files and is plotted for the four selected and four control lines. (PDF 6 kb)

Additional file 6: **Table S4.** Sequencing information for each of the eight pooled RNA libraries. (XLSX 40 kb)

Additional file 7: **Figure S3.** Principal Components Analysis (PCA) of transcriptome-wide expression reveals pervasive line-specific effects of selection on transcriptional variation. The first (two) letter(s) of each line name reflect selection regime (HL = selection, C = control) and the last letter the replicate (A to D). PC 1 (accounting for 23% of variation), separates line pairs A and B from C and D irrespective of selection, while PC 2, 3, 4, and 5 (accounting for 17, 13, 12, and 12% of variation, respectively) capture the effect of selective regime for some line pairs, but not others. PC 6, accounting for 12% of variation, captures the consistent effect of selection across all four line pairs. Correlations between PC 6 and expression of individual transcripts were used to identify transcripts whose expression evolved consistently in response to selection for increased learning ability (see Methods, Fig. 1 and Additional files 5, 7 and 8). (PDF 5 kb)

Additional file 8: **Figure S4.** Genes with consistently evolved expression differences as identified by three separate methods. A total of 36 genes showed significant evolved expression differences consistent across four replicate pairs of lines. We identified these genes as they were significant outliers in differential expression analysis in edgeR, significantly correlated with the axis separating control and selected lines in a PCA, and / or showed significant differences between the selected and control line within line pairs. In addition, we only retained genes with a twofold or higher absolute expression difference as a result of selection (see Methods). (PDF 19 kb)

Additional file 9: **Figure S5.** Evolved expression changes in response to selection for increased learning ability. (a) MA plot showing differential expression between selected and control lines (log_2_ Fold Change), with higher values indicating increased expression in selected lines, plotted as a function of average expression (log_2_ CPM). (b) Volcano plot showing statistical evidence for differential expression from edgeR (−log_10_ *P* value from likelihood ratio test) plotted as a function of differential expression. Significant transcripts identified in edgeR, PCA, or pairwise comparisons are indicated by blue squares, green triangles, and red circles, respectively. (PDF 1899 kb)

Additional file 10: **Table S5.** Evolved expression changes in response to selection for increased learning ability. Thirty-six transcripts, expressed from 34 loci, showed evidence of evolved expression regulation that was consistent across four replicate pairs of lines, as identified by three complementary methods (see Methods and Additional file 8). Transcripts are in order of increasing Fold Change, with high Fold Change indicating elevated expression in lines selected for increased learning ability. Table includes Fold Change per line pair, test statistics for edgeR, correlations with PC 6 and χ^2^ tests within line pairs, chromosomal positions of transcripts, and additional annotations from NCBI and WaspAtlas [43]. (XLSX 53 kb)

Additional file 11: **Figure S6.** Relation between *F*_ST_ and expression divergence (absolute value of log2 Fold Change) for each of the four pairs of selection and control line. Spline curves were generated in R (geom_smooth, span = 0.5). Pearson’s correlations: (a) ρ = 0.02, *P* = 0.004, (b) ρ = 0.008, *P* = 0.36, (c) ρ = 0.02, *P* = 0.02, (d) ρ = 0.03, *P* = 0.0006. (PDF 64 kb)

Additional file 12: **Figure S7.** Gene expression in lines selected for increased learning ability. Heatmap shows absolute expression (log_2_ CPM) in four control lines (left; CA, CB, CC, and CD) and four selected lines (right; HLA, HLB, HLC, and HLD), for 36 significant transcripts, sorted by low to high Fold Change (between selected and control lines). High and low expression are indicated by gold and blue shades, respectively. (PDF 26 kb)

Additional file 13: **Figure S8.** For two genes, alternative transcripts of the same locus have evolved opposite expression patterns in lines selected for increased learning ability. Expression (log_2_ CPM / RPKM) of alternative transcripts of the same locus is plotted in control (C) and selected (HL) lines for *Rho GTPase-activating protein* and for *cap-specific mRNA (nucleoside-2’-O-)-methyltransferase 2*. The two alternative transcripts for each locus are indicated with different colours. (PDF 5 kb)
