# Supplementary figures and images for: Regulatory and sequence evolution in response to selection for improved associative learning ability in Nasonia vitripennis
Source: BMC Genomics. 2018 Dec 10;19:892. doi: 10.1186/s12864-018-5310-9 (PMC6288879; doi:10.1186/s12864-018-5310-9)

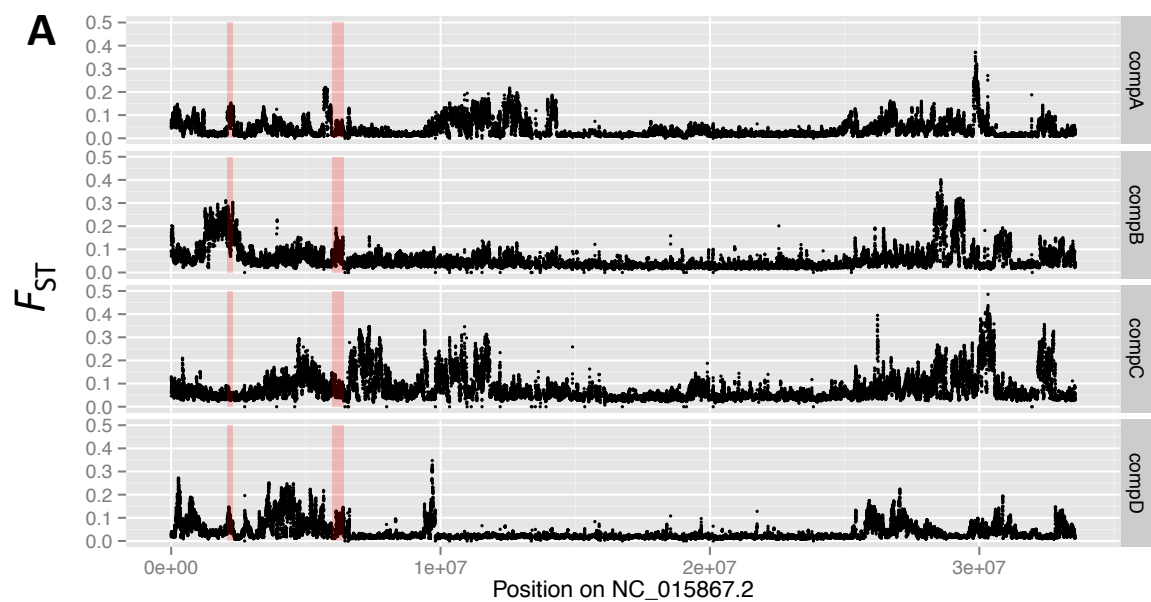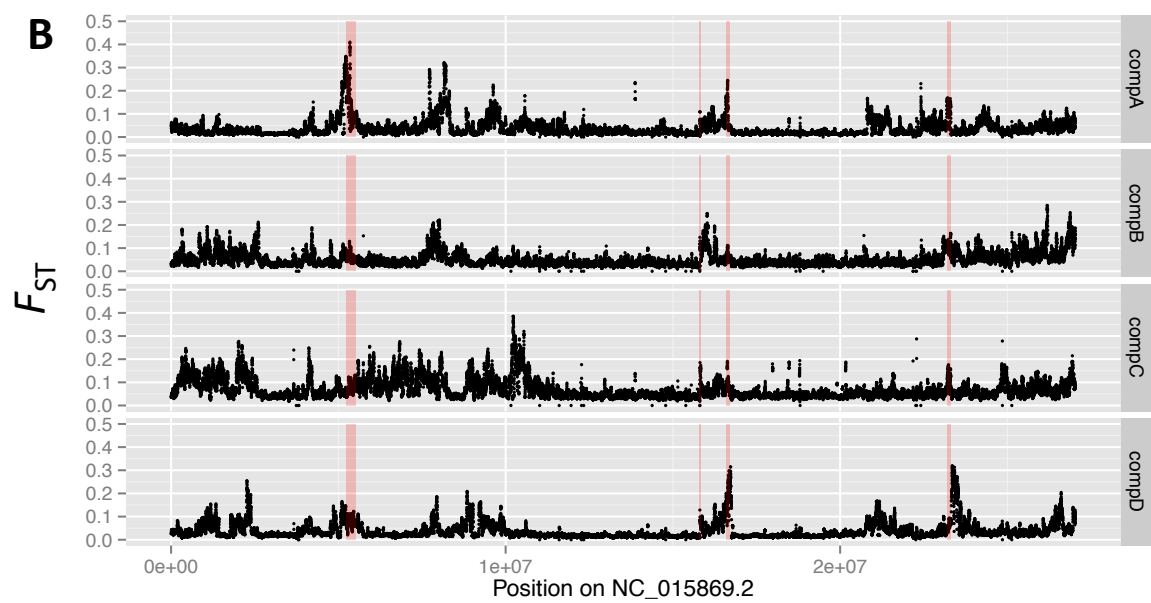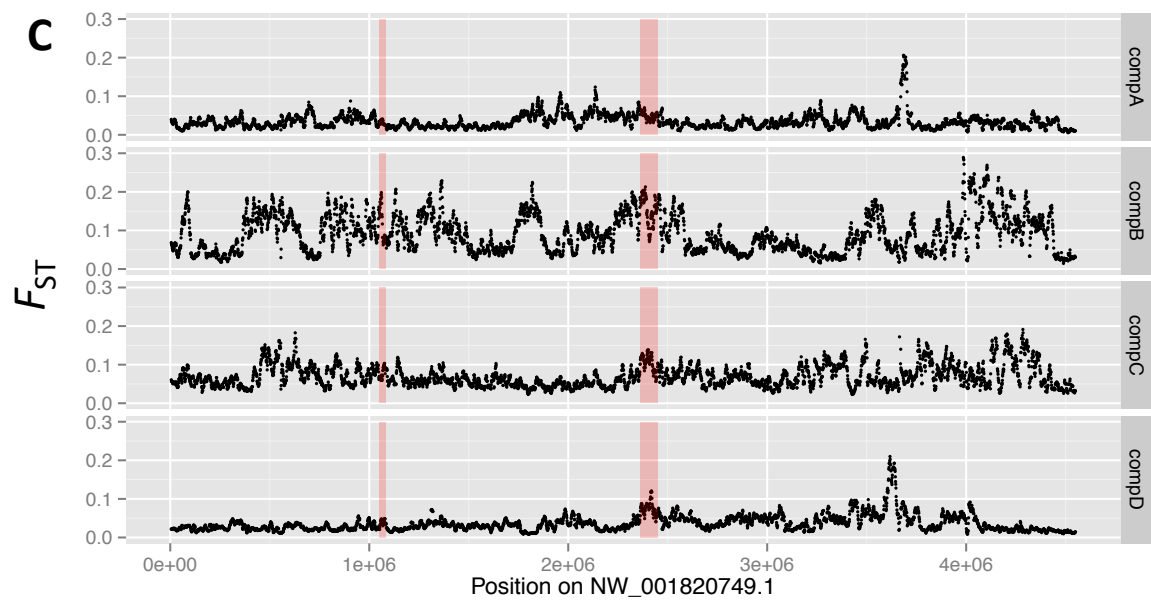

Supplement: Supplementary file 2 — Figure S1. Manhattan plots of FST values for each of the four pairs of selected versus control lines (A, B, C and D). Areas highlighted in red correspond to the clusters of significant SNPs in Fig. 1. (a) Chromosome 1 (scaffold NC_015867.2) (b) Chromosome 2 (scaffold NC_015869.2) (c) unplaced scaffold NW_001820749.1 (note difference in y-axis scaling). (PDF 19568 kb) [file 12864_2018_5310_MOESM2_ESM.pdf]

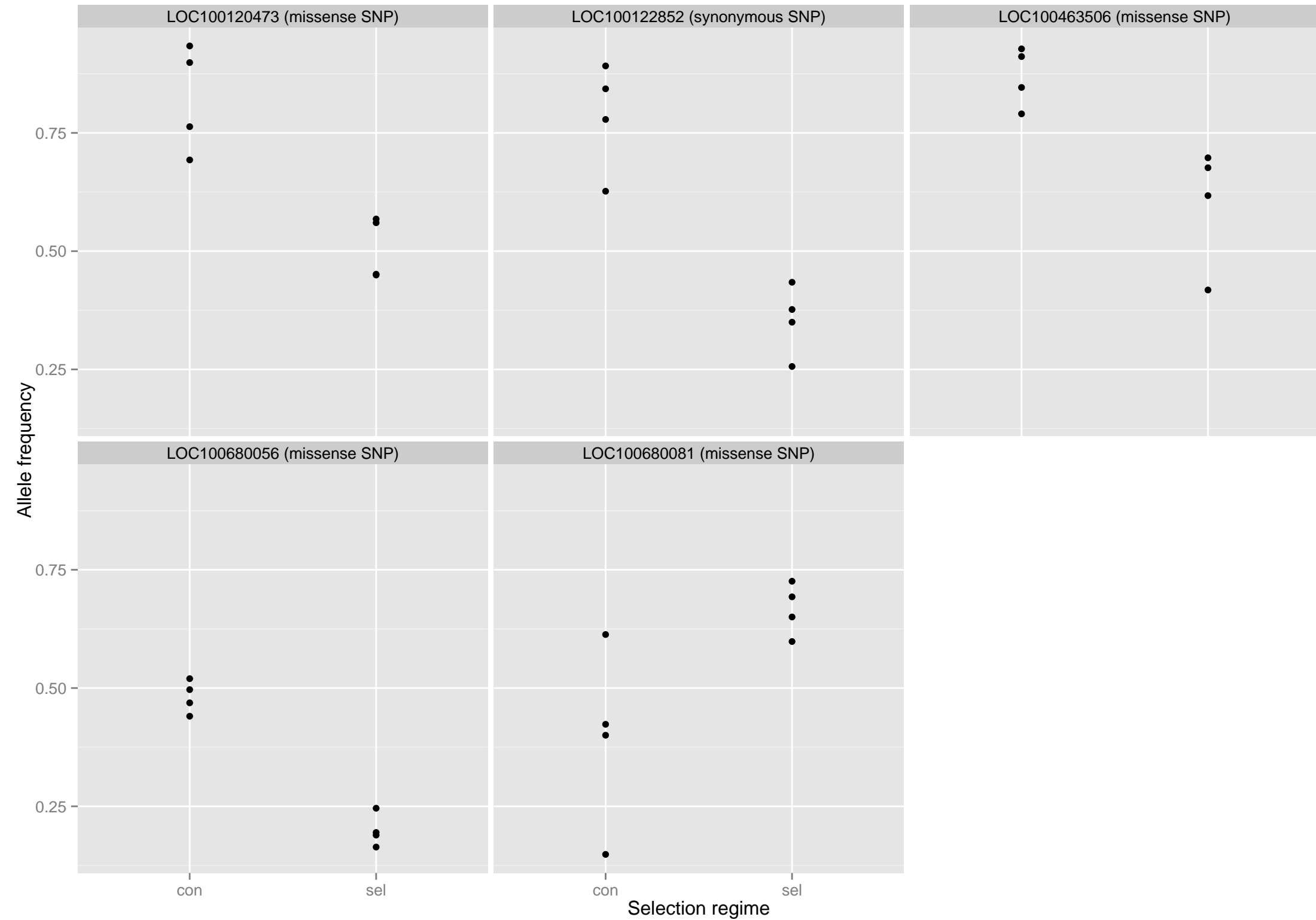

Supplement: Supplementary file 5 — Figure S2. Sanger sequencing validation of five significantly diverged SNPs in coding regions (see Table 1). Frequency of the alternative allele (missense or synonymous) was estimated from the peak height in Sanger sequencing trace files and is plotted for the four selected and four control lines. (PDF 6 kb) [file 12864_2018_5310_MOESM5_ESM.pdf]

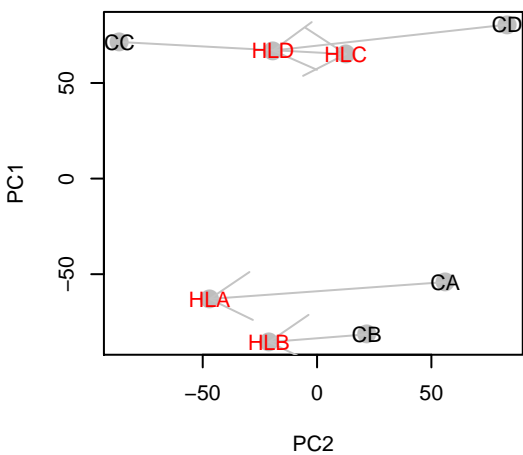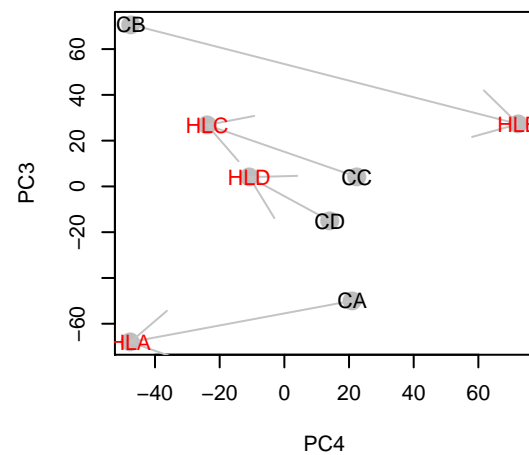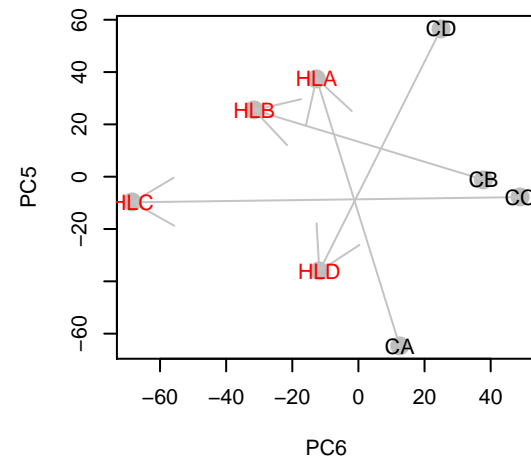

Supplement: Supplementary file 7 — Figure S3. Principal Components Analysis (PCA) of transcriptome-wide expression reveals pervasive line-specific effects of selection on transcriptional variation. The first (two) letter(s) of each line name reflect selection regime (HL = selection, C = control) and the last letter the replicate (A to D). PC 1 (accounting for 23% of variation), separates line pairs A and B from C and D irrespective of selection, while PC 2, 3, 4, and 5 (accounting for 17, 13, 12, and 12% of variation, respectively) capture the effect of selective regime for some line pairs, but not others. PC 6, accounting for 12% of variation, captures the consistent effect of selection across all four line pairs. Correlations between PC 6 and expression of individual transcripts were used to identify transcripts whose expression evolved consistently in response to selection for increased learning ability (see Methods, Fig. 1 and Additional files 5, 7 and 8). (PDF 5 kb) [file 12864_2018_5310_MOESM7_ESM.pdf]

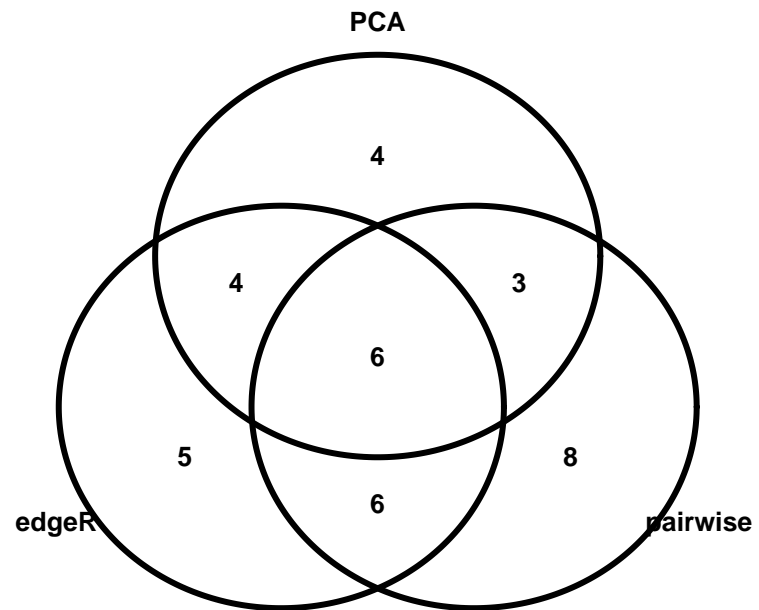

Supplement: Supplementary file 8 — Figure S4. Genes with consistently evolved expression differences as identified by three separate methods. A total of 36 genes showed significant evolved expression differences consistent across four replicate pairs of lines. We identified these genes as they were significant outliers in differential expression analysis in edgeR, significantly correlated with the axis separating control and selected lines in a PCA, and / or showed significant differences between the selected and control line within line pairs. In addition, we only retained genes with a twofold or higher absolute expression difference as a result of selection (see Methods). (PDF 19 kb) [file 12864_2018_5310_MOESM8_ESM.pdf]

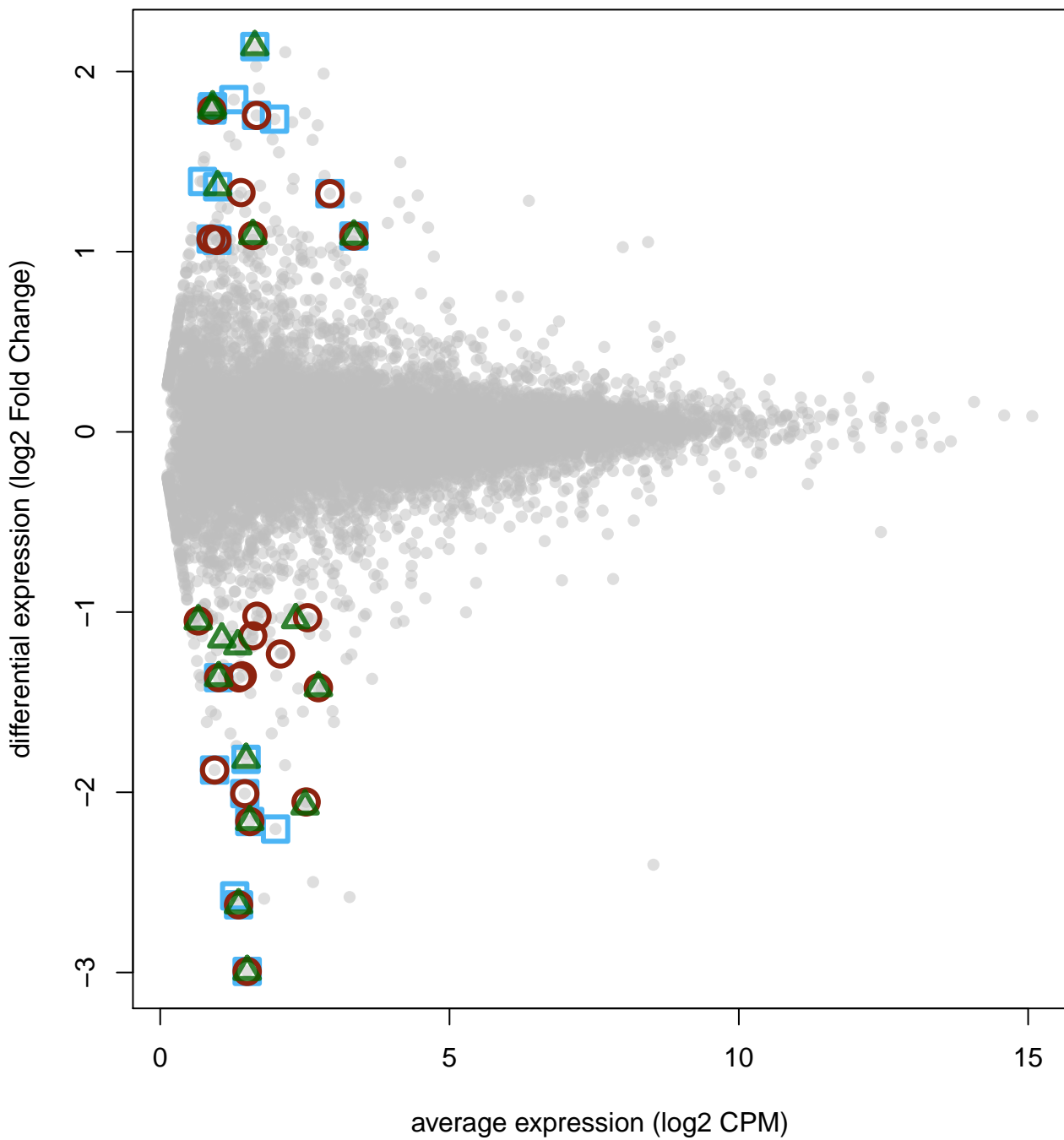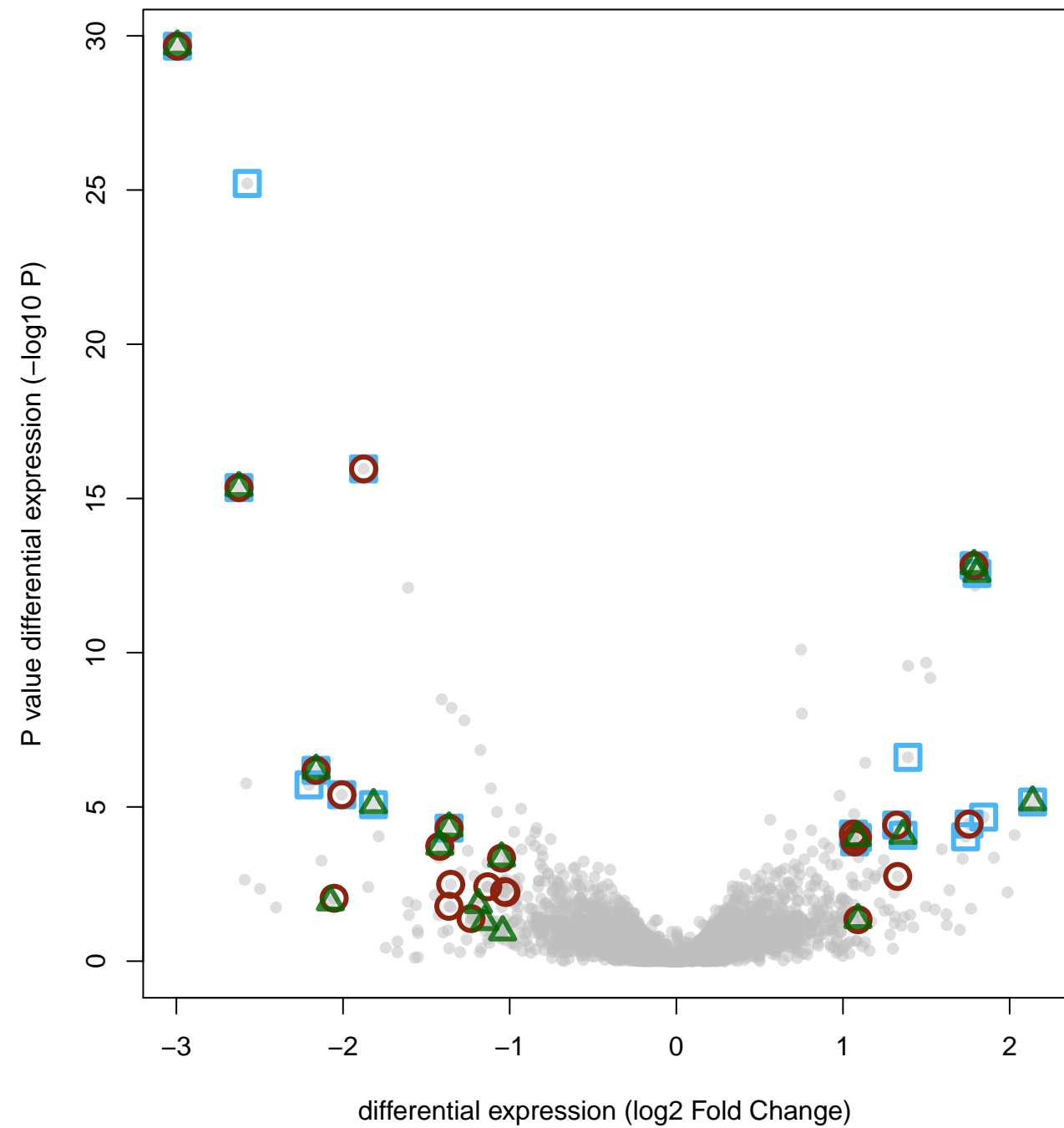

Supplement: Supplementary file 9 — Figure S5. Evolved expression changes in response to selection for increased learning ability. (a) MA plot showing differential expression between selected and control lines (log2 Fold Change), with higher values indicating increased expression in selected lines, plotted as a function of average expression (log2 CPM). (b) Volcano plot showing statistical evidence for differential expression from edgeR (−log10 P value from likelihood ratio test) plotted as a function of differential expression. Significant transcripts identified in edgeR, PCA, or pairwise comparisons are indicated by blue squares, green triangles, and red circles, respectively. (PDF 1899 kb) [file 12864_2018_5310_MOESM9_ESM.pdf]

**A**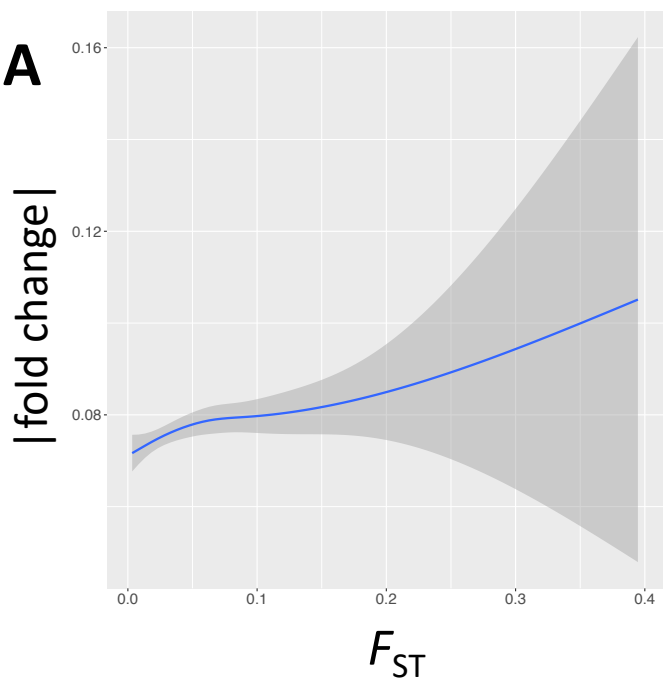**B**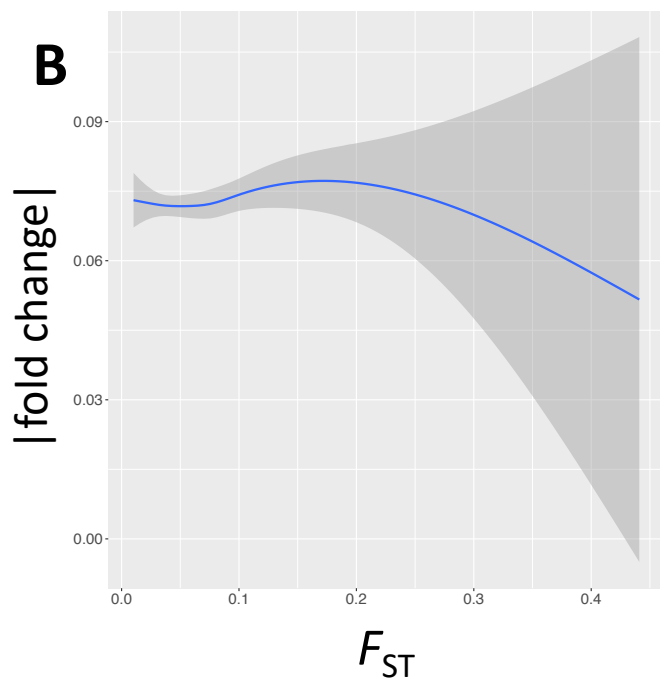**C**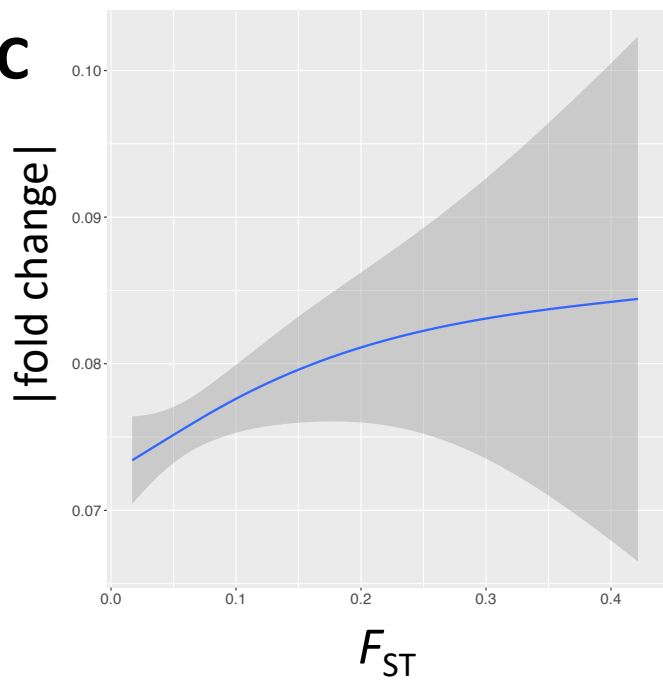**D**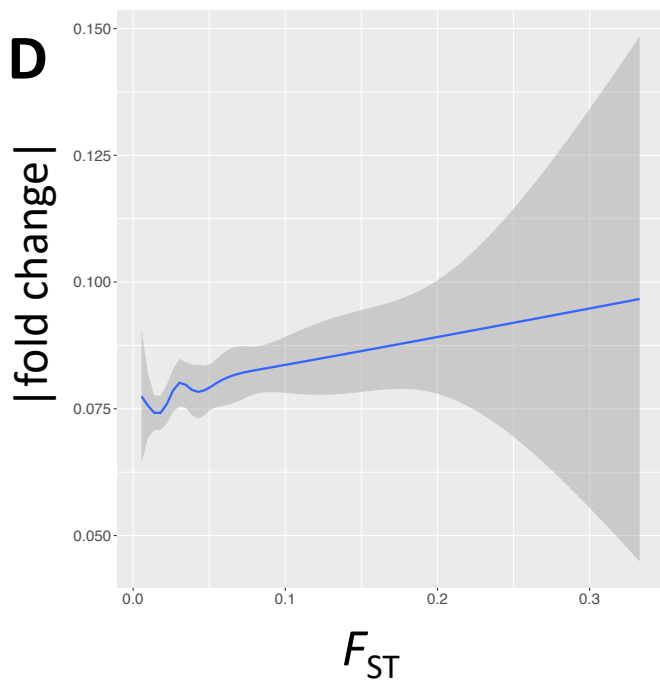

Supplement: Supplementary file 11 — Figure S6. Relation between FST and expression divergence (absolute value of log2 Fold Change) for each of the four pairs of selection and control line. Spline curves were generated in R (geom_smooth, span = 0.5). Pearson’s correlations: (a) ρ = 0.02, P = 0.004, (b) ρ = 0.008, P = 0.36, (c) ρ = 0.02, P = 0.02, (d) ρ = 0.03, P = 0.0006. (PDF 64 kb) [file 12864_2018_5310_MOESM11_ESM.pdf]

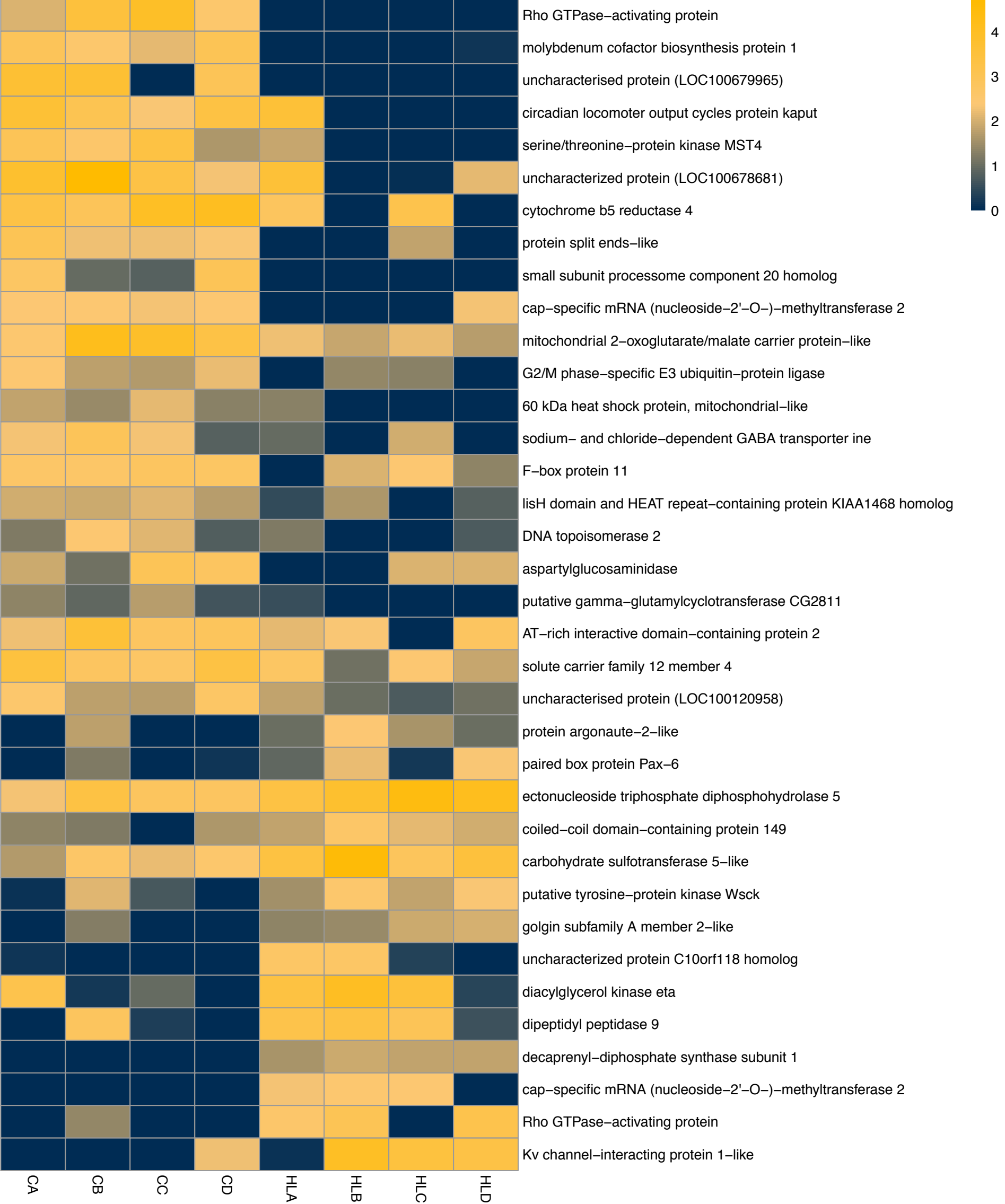

Supplement: Supplementary file 12 — Figure S7. Gene expression in lines selected for increased learning ability. Heatmap shows absolute expression (log2 CPM) in four control lines (left; CA, CB, CC, and CD) and four selected lines (right; HLA, HLB, HLC, and HLD), for 36 significant transcripts, sorted by low to high Fold Change (between selected and control lines). High and low expression are indicated by gold and blue shades, respectively. (PDF 26 kb) [file 12864_2018_5310_MOESM12_ESM.pdf]

Rho GTPase-activating protein

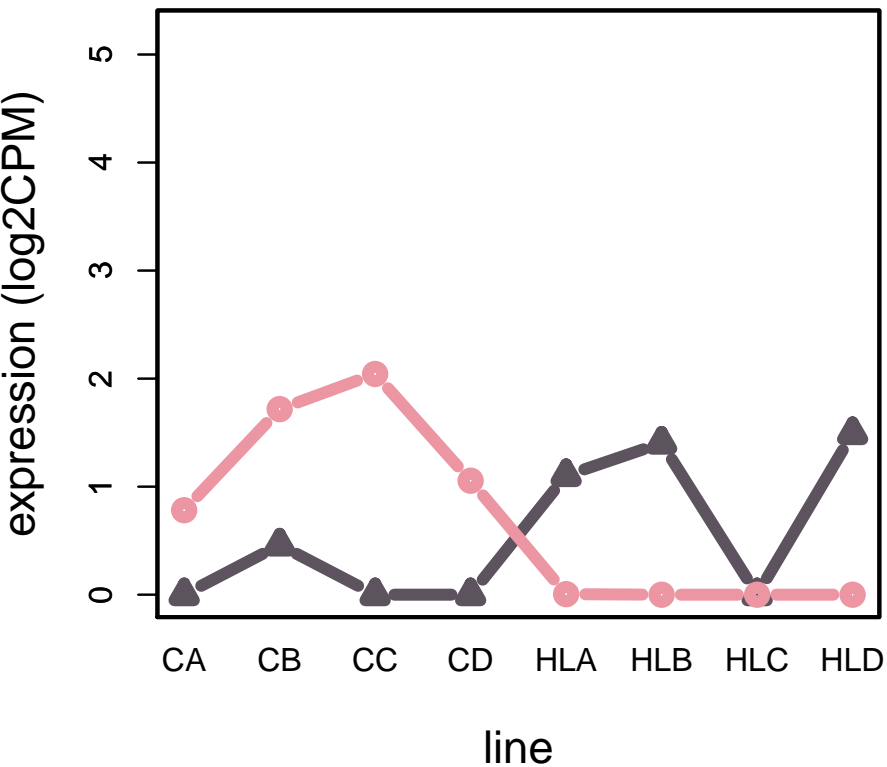

ap-specific mRNA (nucleoside-2'-O-)-methyltransferase

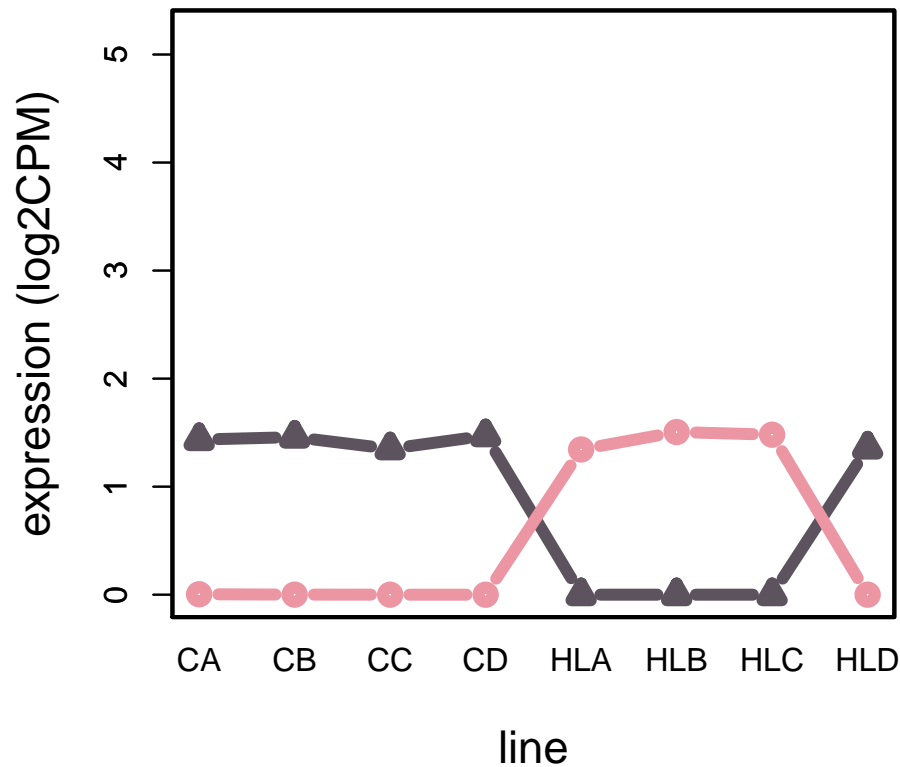

Supplement: Supplementary file 13 — Figure S8. For two genes, alternative transcripts of the same locus have evolved opposite expression patterns in lines selected for increased learning ability. Expression (log2 CPM / RPKM) of alternative transcripts of the same locus is plotted in control (C) and selected (HL) lines for Rho GTPase-activating protein and for cap-specific mRNA (nucleoside-2’-O-)-methyltransferase 2. The two alternative transcripts for each locus are indicated with different colours. (PDF 5 kb) [file 12864_2018_5310_MOESM13_ESM.pdf]
